# Microbiome-Metabolome Analysis of the Immune Microenvironment of the Cecal Contents, Soft Feces, and Hard Feces of Hyplus Rabbits

**DOI:** 10.1155/2022/5725442

**Published:** 2022-10-30

**Authors:** Zhichao Li, Hui He, Mengke Ni, Zhouyan Wang, Chaohui Guo, Yufang Niu, Shanshan Xing, Mingkun Song, Yaling Wang, Yixuan Jiang, Lei Yu, Ming Li, Huifen Xu

**Affiliations:** ^1^College of Animal Science and Technology, Henan Agricultural University, Zhengzhou 450046, China; ^2^Henan University of Chinese Medicine, Zhengzhou 450046, China

## Abstract

The intestinal microbiota and its metabolites play vital roles in host growth, development, and immune regulation. This study analyzed the microbial community distribution and the cytokine and short-chain fatty acid (SCFA) content of cecal contents (Con group), soft feces (SF group), and hard feces (HF group) of 60-day-old Hyplus rabbits and verified the effect of soft feces on the cecal immune microenvironment by coprophagy prevention (CP). The results showed that there were significant differences in the levels of phylum and genus composition, cytokines, and SCFAs among the Con group, SF group, and HF group. The correlation analysis of cytokines and SCFAs with differential microbial communities showed that *Muribaculaceae*, *Ruminococcaceae_UCG-014*, *Ruminococcaceae_NK4A214_group*, and *Christensenellaceae_R-7_Group* are closely related to cytokines and SCFAs. After CP treatment, the contents of propionic acid, butyric acid, IL-4, and IL-10 in cecum decreased significantly, whereas TNF-*α* and IL-1*β* increased significantly. Moreover, the inhibition of coprophagy led to the downregulation of the expression levels of tight junction proteins (Claudin-1, Occludin, and ZO-1) related to intestinal inflammation and intestinal barrier function, and the ring-like structure of ZO-1 was disrupted. In conclusion, coprophagy can not only help rabbits obtain more probiotics and SCFAs but also play an essential role in improving the immune microenvironment of cecum.

## 1. Introduction

Animal feces contain a large number of intestinal microorganisms. Their main function is to digest and absorb nutrients in food and macromolecular substances that the host itself cannot completely decompose, to provide the host with energy and nutrients necessary for growth and development [1]. Carbohydrates are an important energy source in animals. There are a large number of polysaccharides that cannot be decomposed and utilized in the food taken by animals; microorganisms in the intestine provide the host with enzymes and biochemical metabolic pathways that the host lacks so that the host can produce energy by fermenting the degraded polysaccharides (starch, cellulose, hemicellulose, and colloid) and absorbing oligosaccharides. Additionally, a large number of short-chain fatty acids (SCFAs) (acetic acid, propionic acid, and butyric acid) are produced [2–5]. The intestinal microbiota helps to maintain the integrity of the intestinal epithelial mucosal barrier function and causes immune system-mediated mucosal protection. The intestinal microbiota regulates the tight junctions of intestinal epithelial cells and protects intestinal epithelial cells from damage by controlling the proliferation rate of intestinal epithelial cells and inducing cytoprotective proteins [6, 7]. The mucosal epithelial barrier is the first line of defense against the invasion of intestinal pathogenic microorganisms and toxins. As an important part of the intestinal mucosal barrier, changes in tight junction proteins can cause abnormal intestinal barrier function and affect the intestinal health of rabbits, so they play a vital role in the occurrence of intestinal inflammation and diseases. Microbial colonization can stimulate intestinal goblet cells to secrete mucin, which plays a vital role in maintaining intestinal health [8]. In addition, an increasing number of studies have shown that host behavior can affect intestinal microbiota, and intestinal microbiota can also affect animal behavior [9, 10].

Coprophagy refers to the behavior of animals feeding on feces, and animal feeding on feces includes feeding on their own feces and the feces from other animals to meet their nutritional needs [11]. Coprophagy prevention can cause weight loss or growth retardation [12]. Soft feces of rabbits contain a large number of bacterial proteins and nutrients. Eating soft feces can prolong the time of feed passing through the digestive tract, improve the digestion and absorption efficiency of feed, help to maintain the normal microbiota of the digestive tract, and alleviate nutritional diseases [13, 14]. Preventing rabbits from eating their feces reduces the content of vitamin A in the blood, causes them to slowly lose weight, increases the content of acetic acid and propionic acid in the blood, reduces the content of butyric acid, and causes skin damage to the eyes and ears. In addition, fecal eating behavior can also help herbivores obtain the necessary intestinal microbiota and maintain the diversity and function of the intestinal microbiota [15, 16]. Overall, coprophagy plays a very essential role in the nutrition and health in small herbivores.

In recent years, an increasing number of research reports have shown that coprophagy is of great significance to rabbit growth, development, and immune regulation [17, 18]. However, the microbiota and metabolites that play a key role in rabbit soft feces are still unknown. The rapid development of high-throughput sequencing technology has helped researchers better understand the composition of host intestinal microbiota and identify the key microbiota and metabolites, which has played an important role in improving human and animal health [19, 20]. Combined analysis of the microbiome and metabolome can better identify the key regulatory metabolic pathways, determine the key microbiota, and explain the molecular mechanisms of biological growth and development, environmental response, physiological state, and pathological response. Therefore, in this study, high-throughput sequencing technology and gas chromatography-mass spectrometer (GC-MS) were used to analyze the cecal contents, microbial diversity, SCFAs, and cytokines of soft and hard feces from Hyplus rabbits [21]. Subsequently, the effect of soft feces on cecal immune microenvironment was verified by coprophagy prevention treatment in rabbits, aimed to screen the key microbiota and its metabolites that play an important role in regulating the growth, development, and immune function of rabbits.

## 2. Materials and Methods

### 2.1. Animal Ethics

All the experimental procedures applied in this study were reviewed and approved by the Ministry of Science and Technology in China (2014). All procedures involving live rabbit handling, management, and health care are performed under the approval of the Institutional Animal Care and Use Committee (IACUC) of Henan Agricultural University (permit number: 21-0156).

### 2.2. Experimental Design, Animals, and Management

In this study, a total of 32 60-day-old Hyplus rabbits (half male and half female) with similar weight provided by Jiyuan Sunshine Rabbit Technology Co., Ltd. were used as experimental animals. All Hyplus rabbits were fed according to the feeding standard of Jiyuan Sunshine Rabbit Technology Co., Ltd. The composition of the diet is shown in Table 1. After 20 days of adaptive feeding, the soft feces and hard feces of Hyplus rabbits were collected. Eight rabbits were randomly selected for cecal content collection after euthanasia. The collected samples were divided into three groups: cecal content group (Con1-6), soft feces group (SF1-8), and hard feces group (HF1-8). All samples were stored at -80°C for a short time, and DNA was extracted as soon as possible. Then, the remaining 10 Hyplus rabbits were randomly divided into two groups (CN1-5 and CP1-5), wearing collars for the coprophagy prevention (CP) test (half male and half female). In the CP group, rabbits were fitted with a wide collar (7 cm) to prevent them from consuming their feces to build the coprophagy prevention rabbit model; in the CN group, the rabbits were fitted with a narrow collar (2.5 cm) that did not prevent them from consuming their feces. These Hyplus rabbits were kept in cages alone and had free access to feed and water. The same rabbit species (same age), basic dietary composition, feeding method, and environment were used. All rabbits were raised according to the appropriate guidelines for raising rabbits.

### 2.3. Cytokine and SCFA Content Detection in Soft Feces, Hard Feces, and Cecal Contents

An ELISA Kit (Meimian, Jiangsu, China) was used to analyze the content of several cytokines (including TNF-*α*, IL-1*β*, IL-6, TGF-*β*, IL-4, IL-10, and IL-13) in the cecal contents, soft feces, and hard feces. GC-MS (Agilent, CA, USA) was used to detect SCFA content in cecal contents, soft feces, and hard feces, including formic acid, isocaproic acid, heptanoic acid, propanoic acid, isobutyric acid, butyric acid, valeric acid, acetic acid, isovaleric acid, and hexanoic acid.

### 2.4. Extraction and Quality Assessment of Total DNA from Soft Feces, Hard Feces, and Cecal Contents and Library Construction

The fecal genomic DNA Extraction Kit (Tiangen, Beijing, China) was used to extract the fecal genomic DNA from all samples. Meanwhile, NanoDrop was used to quantify DNA, and the quality of DNA was assessed by 1.2% agarose gel electrophoresis. Then, the V3+V4 region of 16S rRNA was amplified by PCR, gel recovery, and fluorescence quantification. The forward primer was 5′-ACTCCTACGGGAGGCAGCA-3′ and the reverse primer was 5′-GGACTACHVGGGTWTCTAAT-3′. Subsequently, the sequencing library was prepared using the TruSeq Nano DNA LT Library Prep Kit, and the library was assessed using an Agilent Bioanalyzer.

### 2.5. Illumina NovaSeq 6000 High-Throughput Sequencing

The qualified library was quantified by the quant-iT PicoGreen dsDNA Assay Kit on the Promega QuantiFluor fluorescence quantitative system. After gradient dilution of qualified online sequencing libraries (index sequences could not be repeated), they were mixed according to the required sequencing amount in the corresponding proportion and denatured into single strands by NaOH for online sequencing. PE250 sequencing was performed with an Illumina NovaSeq 6000 sequencer, and the corresponding reagent was NovaSeq 6000 S2 Reagent Kit v1.5 (300 cycles). Sequencing service was performed by Bioyigene Biotechnology Co., Ltd. (Wuhan, China).

### 2.6. Bioinformatics Analysis of 16S rRNA Gene Sequencing Data

Bioinformatics analysis was mainly based on Xu et al. and Deng et al. [7, 22]. (1) By calculating statistics on the ASV/OTU table after flattening, the specific composition table of the microbial community in each sample at each classification level was obtained, and the column diagram of phylum and genus levels was drawn by QIIME2 software. (2) The ggtree package was used to visualize an evolutionary tree to show the position of each ASV/OTU in the evolutionary tree and the evolutionary distance between them and reflect their composition, abundance, taxonomy, and other information through a heatmap and histogram. (3) In this study, Chao1 and observed species indexes were used to characterize richness, Shannon and Simpson indexes were used to characterize diversity, Faith's PD index was used to characterize evolution-based diversity, Pielou's evenness index was used to characterize evenness, and Good's coverage index was used to characterize coverage. Based on alpha diversity, QIIME2 was used to draw rarefaction curve (reference for calculation method of alpha diversity index http://scikit-bio.org/docs/latest/generated/skbio.diversity.alpha.html#module-Skbio.diversity.alpha). (4) Using the uclust function of stat package in R language, UPGMA algorithm (i.e., average clustering method) was used for clustering analysis of the Bray-Curtis distance matrix by default, and the R package ggtree was used for visualization. (5) The VennDiagram package in R software was used to make a Venn diagram according to the ASV/OTU abundance table, and the number of members of each set was counted according to their presence or absence in each sample (group), that is, the number of unique ASVs/OTUs in each group and the number of common ASVs/OTUs among groups (note that it is not an abundance value). The principal component coordinate scores of each sample and each taxon were calculated by R script, combined with the contents of cytokines and SCFAs, and presented in the form of interaction diagram (RDA figure) (RDA was conducted by using Shanghai Personal Gene Cloud Platform, https://www.genescloud.cn/chart/).

### 2.7. Functional Potential Prediction

The analysis process of PICRUST2 was slightly modified on the basis of Langille et al. [23]. Its important function is to count the metabolic pathway of the microbiota and analyze the differences in metabolic pathways and the species composition of metabolic pathways. The main analysis process was as follows: (1) first, the 16S rRNA gene sequence of the known microbial genome was aligned to construct a new evolutionary tree. (2) Using the castor hidden state prediction algorithm, according to the copy number of the gene family corresponding to the reference sequence in the evolutionary tree, the nearest sequence species of the characteristic sequence was inferred, and then, the copy number of gene family was obtained. (3) The copy number of the gene family of each sample was calculated by combining the abundance characteristic sequences of each sample. (4) Finally, the gene family was “mapped” to various databases, and MinPath was used to infer the existence of metabolic pathways to obtain the abundance data of metabolic pathways in each sample. (5) After obtaining the abundance data of metabolic pathways, the metagenomeSeq package in R software was used to determine the metabolic pathways with significant differences between groups. (6) The functional composition of samples/groups was obtained, and the species composition of the pathways was analyzed by using the hierarchical sample metabolic pathway abundance table (Table S2).

### 2.8. Correlation Analysis of Cytokines, SCFAS, and Microbiota

In order to analyze the correlation (Pearson correlation) between the microbiota, cytokines, and SCFAs in the three group, we used OmicShare Tools (Pearson correlation analysis was performed using the OmicShare tools, a free online platform for data analysis (https://www.omicshare.com/tools)) online analysis software to analyze the correlation between the first 15 genera and the cytokines and SCFAs in the Con group, HF group, and SF group. The cytokines include TNF-*α*, IL-1*β*, IL-6, TGF-*β*, IL-4, IL-10, and IL-13; the short-chain fatty acids included formic acid, isocaproic acid, heptanoic acid, propanoic acid, isobutyric acid, butyric acid, valeric acid, acetic acid, isovaleric acid, and hexanoic acid.

### 2.9. Verification the Regulatory Function Soft Feces in Cecal Immune Microenvironment by Coprophagy Prevention Model

ELISA kits were used to detect the contents of TNF-*α*, IL-1*β*, IL-4, and IL-10 in the cecal content of CN and CP groups. GC-MS was used to detect acetic acid, propionic acid, and butyric acid in cecal contents of CP and CN groups. Subsequently, the cecal tissues in the CN and CP groups were subjected to HE and immunofluorescence staining to analyze the protein expression levels of ZO-1. In addition, the mRNA and protein expression levels of *ZO-1*, *OCLN*, and *CLDN1* were measured by qRT-PCR and Western blot, respectively. The Western blot antibody was purchased from Servicebio (Item No. ZO-1: GB111402; Claudin-1: GB112543; Occludin: GB111401). The preparation of paraffin sections refers to Miao et al. [24]. After the sections are prepared, they were placed in a pathology section scanner (Hungary, 3DHISTECH) to collect images with the following parameters: DAPI UV excitation wavelength 330-380 nm, emission wavelength 420 nm, and blue light; CY5 was set to pink light.

### 2.10. Statistical Analysis

A completely randomized trial design was used in this study. The two groups were statistically analyzed by Student's *t* test and one-way ANOVA. In high-throughput sequencing, the statistical method between two groups was Student's *t* test, and three groups or more was one-way ANOVA. An ^∗^ indicates significant difference, *P* < 0.05; ^∗∗^ indicates a very significant difference, *P* < 0.01; NS indicates that there is no significant difference between the data, i.e., *P* > 0.05.

## 3. Results

### 3.1. Detection Results of Cytokines in Soft and Hard Feces and Cecal Contents

**Figure 1:**
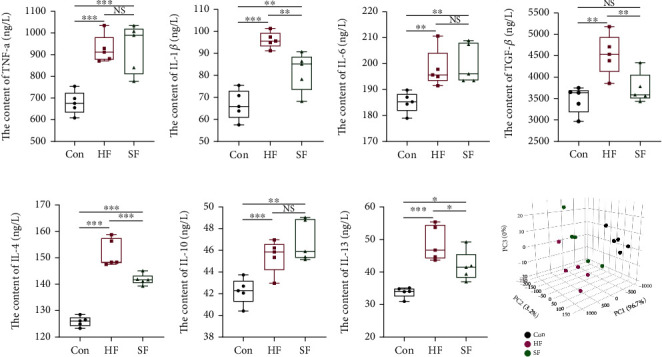
Cytokine content in cecal contents and soft and hard feces. (a–g) The content of cytokines such as TNF-*α*, IL-1*β*, IL-6, TGF-*β*, IL-4, IL-10, and IL-13, respectively, in the cecal contents and soft and hard feces. (h) The three-dimensional PCA diagram of cytokine content in normalized cecal contents and soft and hard feces.

TNF-*α*, IL-1*β*, IL-6, TGF-*β*, IL-4, IL-10, and IL-13 were significantly higher in both the soft and hard feces groups than in the cecal content group; IL-1*β*, TGF-*β*, IL-4, and IL-13 contents were higher in the hard feces group than in the soft feces group, and the difference was statistically significant between the two groups (Figures 1(a)–1(g)). The results of PCA showed that there was less dispersion between samples within groups in the cecal content group, and the dispersion between samples within groups in the soft feces and hard feces groups was greater than that in the cecum content group; the samples between the cecum content group, soft feces group, and hard feces group were each independently distributed, with the samples in the soft and hard feces groups being closer together and farther away from the samples of the cecum content group (Figure 1(h)).

### 3.2. Detection Results of SCFAs in Soft Feces, Hard Feces, and Cecal Contents

**Figure 2:**
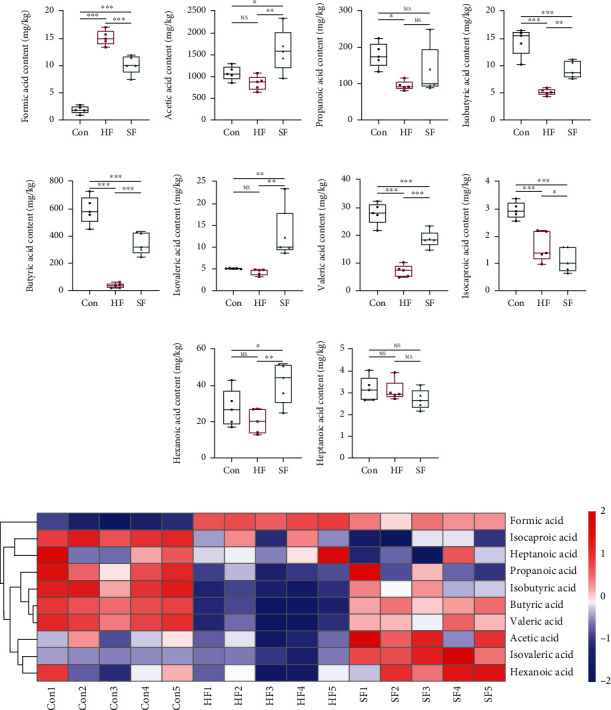
SCFA Content in cecal contents and soft and hard feces. (a–j) The contents of SCFAs such as formic acid, isocaproic acid, heptanoic acid, propanoic acid, isobutyric acid, butyric acid, valeric acid, acetic acid, isovaleric acid, and hexanoic acid in cecal contents and soft and hard feces, respectively. (k) The relative abundance of SCFAs such as formic acid, isocaproic acid, heptanoic acid, propanoic acid, isobutyric acid, butyric acid, valeric acid, acetic acid, isovaleric acid, and hexanoic acid in cecal contents and soft and hard feces in the form of heatmap.

The GC-MS method was used to determine the content of volatile short-chain fatty acids in the three groups, and the results are shown in Figure 2. The contents of isobutyric acid, butyric acid, valeric acid, and isohexanoic acid in the cecal content group were significantly higher than those in the soft feces group and hard feces group (*P* < 0.05). The contents of acetic acid, isobutyric acid, butyric acid, isovaleric acid, valeric acid, and caproic acid in the soft feces group were significantly higher than those in the hard feces group (*P* < 0.05). The content of formic acid content in the soft feces group and the hard feces group was significantly higher than that of the cecal content group (*P* < 0.05), and the content of the hard feces group was higher than that of the soft feces group; the difference was statistically significant (*P* < 0.05). There was no significant difference in the content of enanthic acid in the cecal content group, soft feces group, and hard feces group (*P* > 0.05).

### 3.3. Species Composition among Soft Feces, Hard Feces, and Cecal Content Groups

**Figure 3:**
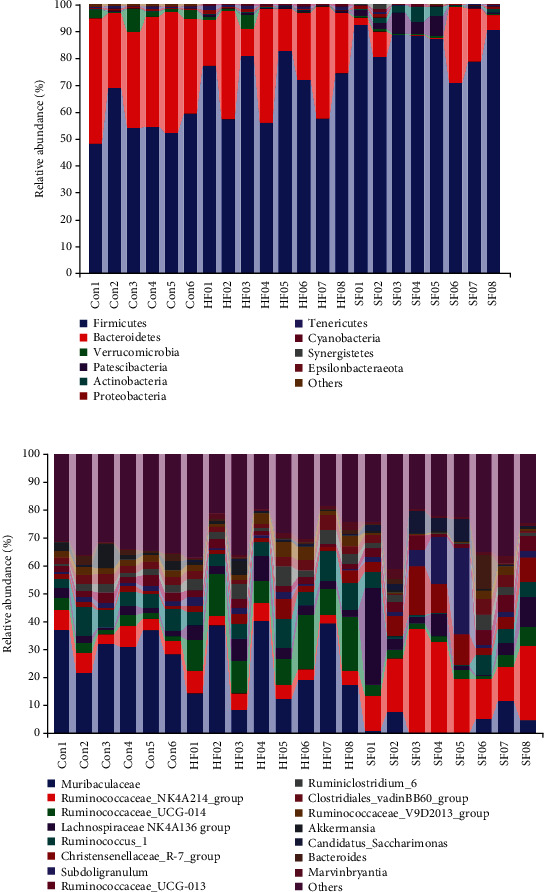
Microbiota composition of cecal contents and soft and hard feces. (a) Composition and distribution of microbiota of cecal contents and soft and hard feces at the phylum level. (b) Composition and distribution of microbiota of cecal contents and soft and hard feces at genus level.

Using QIIME software, the composition and abundance distribution of each sample at both the phylum and genus levels were obtained, and the analysis results were represented in the form of a histogram. At the phylum classification level (Figure 3(a)), Firmicutes and Bacteroides were the main phyla of the three groups of samples. The relative abundance of Firmicutes (84.48%) in the soft feces group was significantly higher than that in the hard feces group (69.66%) and cecal content group (56.82%), but the relative abundance of Bacteroides in the soft feces group (8.30%) was significantly lower than that in the hard feces group (26.67%) and cecal content group (38.31%). At the genus level (Figure 3(b)), the relative abundance of *Muribaculaceae* in the cecal content group (31.17%) and the hard feces group (23.74%) was significantly higher than that of the soft feces group (3.78%) and *Ruminococcaceae_NK4A214_group* in the soft feces group. The relative abundances of *Lachnospiraceae_NK4A136_group*, *Christensenellaceae_R-7_group*, and *Subdoligranulum* (21.73%, 8.18%, 8.13%, and 7.93%, respectively) in the soft feces group were significantly higher than those of the cecal content group (5.71%, 2.30%, 1.39%, and 1.56%, respectively) and hard fecal group (5.07%, 4.76%, 2.92%, and 1.34%, respectively); the relative abundance of *Ruminococcaceae_UCG-014* (12.84%) in the hard feces group was significantly higher than that of the cecal content group (2.80%) and soft feces group (3.39%).

### 3.4. Alpha and Beta Diversity of Microbiota in Soft Feces, Hard Feces, and Cecal Content Groups

**Figure 4:**
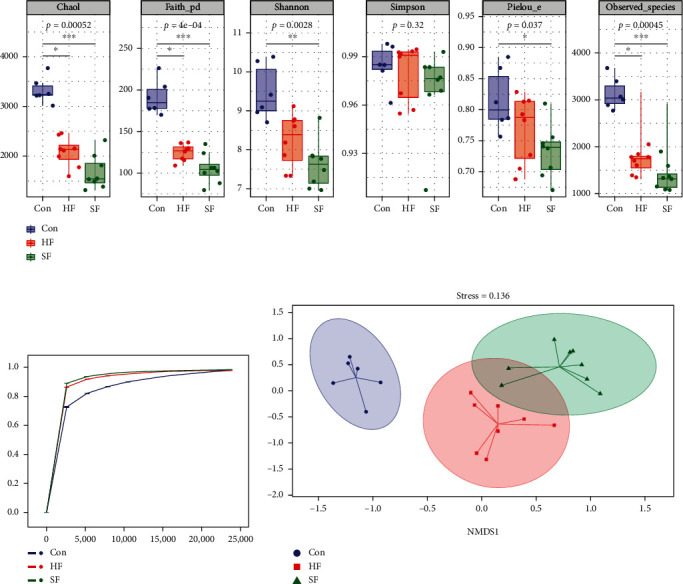
Alpha and beta diversity of cecal contents and soft and hard feces. (a) Alpha diversity of Chao1, observed species, Shannon, Simpson, Faith's PD, and Pielou's evenness in cecal contents and soft and hard feces. (b) Good's coverage sparse curve reflecting the sequencing depth of 16S rRNA gene sequencing. (c) PCoA analysis results of cecal contents and soft and hard feces.

The alpha diversity results are shown in Figure 4(a). The Chao1, Faith's PD, Shannon, Pielou's evenness, and observed species in cecal content group were significantly higher than those in the soft feces group and hard feces group. There was no significant difference in any of the above indices between the soft feces group and the hard feces group (*P* > 0.05). The sparse curve results of Good's coverage are shown in Figure 4(b). The smoothness of the curves of the three groups of samples reflects the impact of sequencing depth on the diversity of observed samples. The curves of the three groups of samples tended to be gentle, indicating that the sequencing results sufficiently reflected the diversity contained in the current samples. If the sequencing depth had continued to increase, it would have indicated that a large number of new ASV/OTUs had not been found. Then, the dimension of multidimensional microbial data was reduced by principal coordinate analysis (PCoA), and the main trend of data change is displayed by the distribution of samples on the continuous sorting axis. In addition, permutational multivariate analysis of variance (PERMANOVA) was used to identify whether there were significant differences among the three groups of samples. The beta diversity of the three groups of samples was analyzed by the above methods. The results showed that in the PCoA diagram (Figure 4(c)), there was an obvious separation between the samples of the cecal content group, the soft feces group, and the hard feces group, and the dispersion between the samples of the cecal content group was less than that between the samples of soft feces group and hard feces group. The results of difference analysis between groups are shown in Figure 4(c). There was a very significant difference between the cecal content group and the soft and hard feces group (*P* < 0.05), but there is no significant difference between the soft and hard feces groups (*P* > 0.05).

### 3.5. Species Differences and Marker Analysis

**Figure 5:**
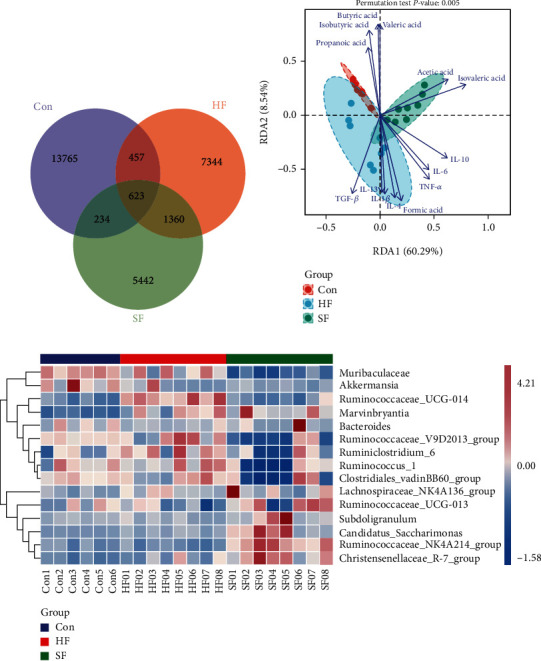
Different species and markers of cecal contents and soft and hard feces. (a) The Wayne diagram drawn by ASV/OUT in three groups of samples of cecal content group and soft and hard feces group. (b) The RDA diagram of correlation analysis between samples of cecal content group and soft and hard feces group and cytokines and SCFAs. (c) Relative abundance of the top 15 bacteria in the cecal content group and soft and hard feces group.

The ASV/OTU abundance table (Table S1) was used to make a Venn diagram of the Con group, the SF group, and the HF group. The three groups of samples had 13,765, 5,442, and 7,344 ASV/OTUs, respectively. Three groups of samples had a total of 623 ASV/OUTs (Figure 5(a)). The RDA results showed that SCFAs were mainly correlated to the Con group and SF group, and cytokines were mainly correlated with the fecal microbiome (HF and SF groups) (Figure 5(b)). The relative abundance of the top 15 bacterial genera in the three groups of samples was drawn using software as shown in Figure 5(c). The relative abundance of *Muribaculaceae* and *Ruminococcus_1* in the *Muribaculaceae* Con group and the HF group was higher than that in the SF group and Ruminococcaceae_NK4A214_group in the SF group. The relative abundances of *Christensenellaceae_R-7_group*, *Subdoligranulum*, and *Candidatus_Saccharimonas* in the SF group were higher than those in the Con group and the HF group.

### 3.6. Functional Potential Predictive Analysis

**Figure 6:**
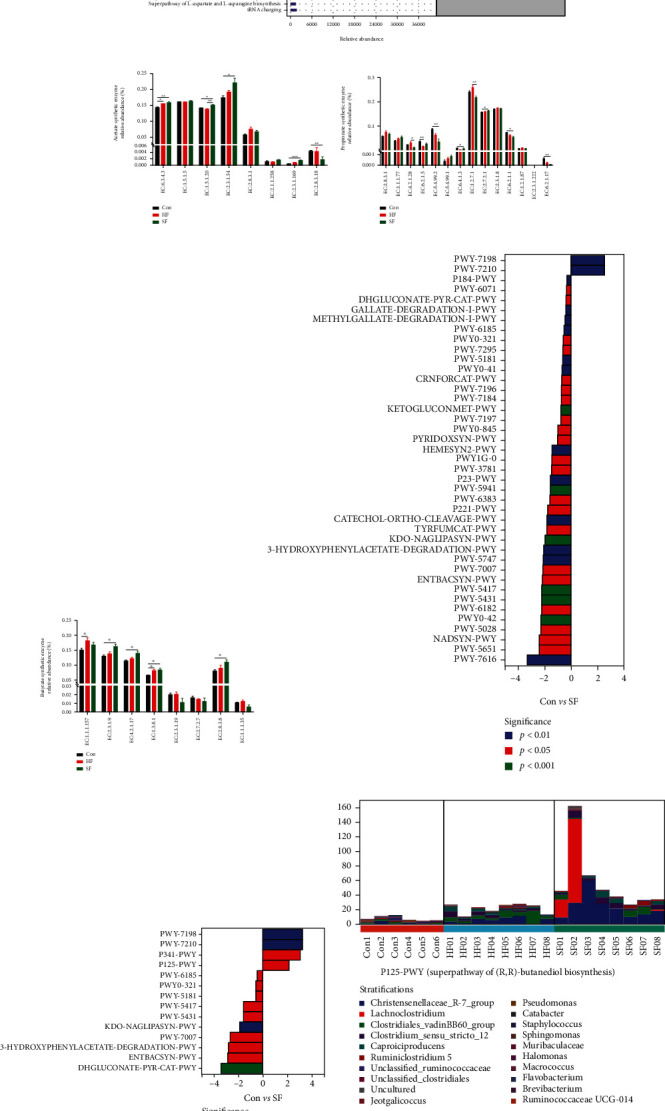
Functional potential prediction of microbiota in cecal contents and soft and hard feces. (a) The analysis results of the MetaCyc metabolic pathway including various pathways involved in primary and secondary metabolism and related metabolites, biochemical reactions, enzymes, and genes. (b) The relative abundance of enzymes related to acetic acid synthase in the cecal content group and soft and hard feces group. (c) The relative abundance of propionate synthase-related enzymes in the cecal content group and soft and hard feces group. (d) The relative abundance of butyrate synthase-related enzymes in cecal content group and soft and hard feces group. (e) Different metabolic pathway between cecal content group and hard fecal group. (f) Different metabolic pathway between cecal content group and soft feces group. (g, h) The species composition of upregulated metabolic pathways such as P341-PWY and P123-PWY in soft feces group compared with the hard feces group.

The relative abundance results of primary and secondary metabolic pathways in the MetaCyc database are shown in Figure 6(a). The relative abundance of pathways related to biosynthesis was high, mainly including amino acid biosynthesis, carbohydrate biosynthesis, coenzyme factor, and vitamin biosynthesis. The relative abundances of enzymes related to the synthesis of acetic acid, propionic acid, and butyrate in the three groups were further analyzed. The relative abundances of acetic acid synthase such as EC: 6.3.4.3, EC: 1.5.1.20, EC: 2.3.1.54, and EC: 2.3.1.169 in the soft feces group were significantly higher than those in the Con group (Figure 6(b)). For propionate synthase, the relative abundance of the EC: 2.7.1.1 enzyme in the soft feces group was significantly higher than that in the Con group (Figure 6(c)). Among butyrate synthases, the relative abundances of EC: 2.3.1.9, EC: 4.2.1.17, EC: 1.3.8.1, and EC: 2.8.3.8 in the soft feces group were significantly higher than those in the Con group (Figure 6(d)). After obtaining the abundance data of metabolic pathways, metagenomeSeq was used to figure out the metabolic pathways with significant differences among the three groups. As shown in Figures 6(e) and 6(f), the upregulated metabolic pathways in the hard fecal group were PWY-7198 and PWY-7210 compared with the cecal content group, and the upregulated metabolic pathways in the soft fecal group were PWY-7198, PWY-7210, P341-PWY, and P123-PWY compared with the cecal content group; that is, P341-PWY and P123-PWY were upregulated in soft feces group relative to hard feces group. Finally, according to the significantly different metabolic pathways, the hierarchical sample metabolic pathway abundance table (Table S2) is used to analyze the species composition of different pathways. The results are shown in Figures 6(g) and 6(h). The P125-PWY pathway is superpathway of (R,R)-butanediol biosynthesis, in which the relative abundance of *Christensenellaceae_R-7_group* in the soft feces group is higher than that of the cecal content group and the hard feces group; the P341-PWY pathway is glycolysis V (Pyrococcus), of which the soft feces group and the hard feces group. The relative abundance of *Lachnoclostridium* in the group was higher than that of the cecal content group.

### 3.7. Correlation Analysis of Cytokines, SCFAs, and Microbiota

**Figure 7:**
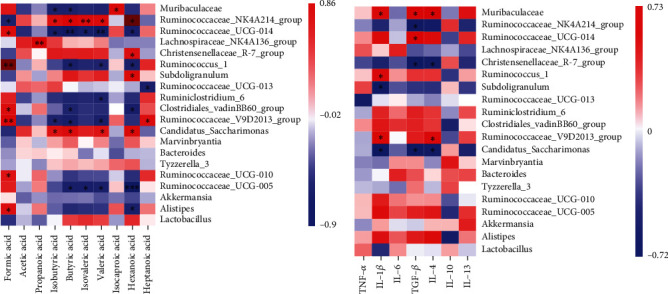
Correlation analysis between SCFAs, cytokines, and different microbiota. (a) The correlation analysis between the bacteria with the top 15 relative abundance and SCFAs. (b) Correlation analysis between the bacteria with the top 15 relative abundance and cytokines.

The correlation analysis results of SCFAs and the microbiota are shown in Figure 7(a). Among them, *Ruminococcaceae_NK4A214_group* had a significant positive correlation with acetic acid, butyric acid, isobutyric acid, isovaleric acid, and hexanoic acid; *Christensenellaceae_R-7_group* had a positive correlation with acetic acid, butyric acid, isobutyric acid, isovaleric acid, and hexanoic acid; *Muribaculaceae* has a significant positive correlation with isocaproic acid; and *Ruminococcaceae_UCG-014* had a significant negative correlation with acetic acid, propionic acid, isobutyric acid, butyric acid, valeric acid, and caproic acid. The results of the correlation analysis between cytokines and the top 15 most abundant bacteria in the three groups of samples are shown in Figure 7(b). *Muribaculaceae* has a significant positive correlation with IL-1*β*, IL-4, and TGF-*β*; in contrast, *Candidatus_Saccharimonas* has a significant negative correlation with IL-1*β*, IL-4, and TGF-*β*.

### 3.8. Effect of Coprophagy Prevention on the Cecal Immune Microenvironment

**Figure 8:**
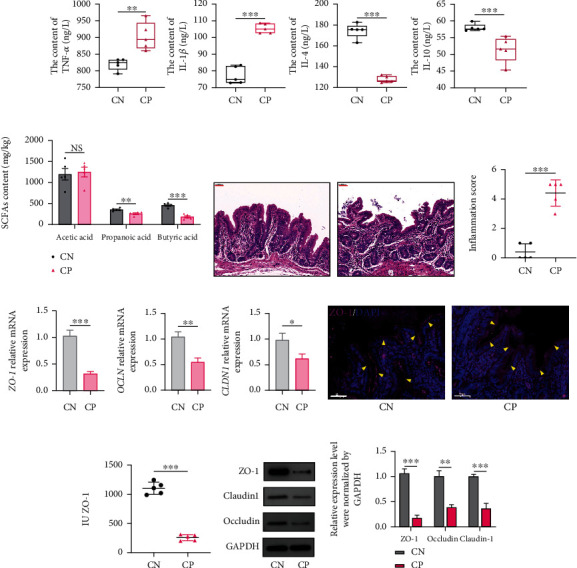
Effect of coprophagy prevention on cytokines and tight junction proteins in cecal contents. (a) Changes in cytokines such as TNF-*α*, IL-1*β*, IL-4, and IL-10 in the contents of the cecum after coprophagy prevention. (b) Changes in SCFAs in cecal contents after coprophagy prevention. (c) HE staining. (d) Immunological score analysis and staining results. (e) The mRNA expression levels of *ZO-1*, *OCLN*, and *CLDN1*. (f) Immunofluorescence staining to analyze the changes in the expression levels of ZO-1. (g) Fluorescence analysis results of ZO-1. (h) WB analysis of ZO-1, Claudin-1, and Occludin protein expression levels. (i) Grayscale analysis of ZO-1, Claudin-1, and Occludin protein expression levels.

After coprophagy prevention, the cytokines TNF-*α* and IL-1*β* significantly increased, whereas the cytokines IL-4 and IL-10 significantly decreased (Figure 8(a)). After coprophagy prevention, the contents of propionic acid and butyric acid were significantly lower than those in the control group (*P* < 0.01 or *P* < 0.001), and there was no significant difference in the content of acetic acid between the two groups (*P* > 0.05) (Figure 8(b)). HE staining showed that the intestinal mucosa in the CP group was damaged (Figure 8(c)). In addition, based on the results of HE staining, the intestinal samples of the CN group and CP group were scored for inflammation (Figure 8(d)). The results showed that the inflammation score of the CP group was significantly higher than that of the CN group (*P* < 0.001). qRT-PCR results showed that the mRNA expression levels of *ZO-1*, *OCLN*, and *CLDN1* in the CP group were significantly lower than those in the CN group (*P* < 0.05 or *P* < 0.01 or *P* < 0.001) (Figure 8(e)). The staining degree of ZO-1 protein (purple) in the CP group was shallower (compared with that in the CN group), indicating that the abundance of ZO-1 protein in the CP group was lower (Figures 8(f) and 8(g)). Moreover, the Western blot results were consistent with the qRT-PCR results. The protein expression levels of ZO-1, Occludin, and Claudin1 in the CP group were significantly lower than those in the CN group (*P* < 0.01 or *P* < 0.001) (Figures 8(h) and 8(i)).

## 4. Discussion

Due to the special colon separation mechanism of rabbits, they can produce two different forms of feces: soft feces and hard feces [25, 26]. There are many nutrients in soft feces, and rabbits begin eating soft feces at a very early age. This behavior aids in prolonging the retention time of food in the digestive tract and improving the utilization rate of nutrients in food [27–29]. The chemical composition of soft feces is similar to that of the cecal contents, and there are a large number of microorganisms in both of them. These microorganisms can produce a variety of enzymes and SCFAs and play an important role in improving rabbit growth performance and immune function [5, 30]. In this study, the combination analysis of microbiome and metabolome was performed to explore the difference in microbial diversity, SCFAs, and cytokine concentration of cecal contents, soft feces, and hard feces in Hyplus rabbit; our results help better understand the key regulatory metabolic pathways, key flora, and their metabolites and play an important role in clarifying the molecular mechanism of soft feces in rabbit growth, intestinal development, and immune regulation.

The value of Firmicutes/Bacteroides may be related to obesity and emaciation in the host [31–33]. In this study, the relative abundance of *Muribaculaceae* in the Con group and the HF group was higher than that in the SF group at the genus classification level. The relative abundance of this genus brings about a very important impact on host intestinal development and health. The increase in *Akkermansiaceae* and the decrease in *Muribaculaceae* can promote the healthy development of bones [34]. Additionally, *Akkermansiaceae*, especially *Akkermansia muciniphila*, plays a protective role in diet-induced obesity and other diseases [35]. Moreover, the relative abundance of the specific genus *Ruminococcaceae_NK4A214_group* was reduced in the HF group, and it was significantly correlated with bile acid and vitamin A levels, which is proved to be vital for the treatment of rats with ulcerative colitis [36]. *Christensenellaceae_R-7_group* as a probiotic is significantly negatively correlated with body mass index (BMI) and inflammation and metabolic diseases such as IBD and metabolic syndrome [37]. Through association analysis (microbiome and metabolomics analysis), it was confirmed that *Muribaculaceae*, *Christensenellaceae_R-7_group*, and *Ruminococcaceae_NK4A214_group* have a strong positive correlation with SCFAs and IL-10, and they have a strong negative correlation with TNF-*α*, IL-1*β*, and TGF-*β*. *Candidatus_Saccharimonas* has a strong negative correlation with IL-1*β*, TGF-*β*, and IL-4. These results show that there are abundant genes (microorganisms) that can synthesize SCFAs (acetic acid, propionic acid, and butyric acid) in soft feces, which may be the key for promoting growth and development and resisting external environmental interference [38, 39]. These findings may also help explaining why the host can improve the digestion and absorption efficiency of feed and help uncovering the underlying mechanism that eating soft feces helps maintaining the normal microbiota of the digestive tract and alleviates nutritional diseases.

After coprophagy prevention, the content of IL-4 and IL-10 in the cecal contents decreased, and the content of TNF-*α* and IL-1*β* increased, which further verified that some microorganisms in soft feces can increase the expression level of cytokines associated with anti-inflammation and reduce the expression level of cytokines involved in the process of inflammation. The intestinal mucosa is a physical barrier against invading intraluminal pathogens and toxins [40, 41]. The results of immunofluorescence staining further confirmed that coprophagy prevention caused damage to the intestinal mucosa, indicating the importance of soft feces in maintaining the intestinal immune microenvironment. Moreover, HE and immunofluorescence staining showed that intestinal inflammation occurred, the ring structure of ZO-1 was destroyed, and the expression of tight junction associated proteins decreased significantly after coprophagy prevention. The tight junctions (TJs) mainly maintain the integrity of the epithelial barrier by regulating the paracellular permeability [42]. The expression level of intestinal TJ proteins is closely related to the integrity of the intestinal barrier, which has an important impact on the balance of the intestinal environment and even body health. In the process of coprophagy prevention, the body suddenly interrupted the intake of probiotics, SCFAs, and cytokines in the external environment. Over time, the homeostasis of the intestinal environment is disrupted, leading to diseases such as enteritis and intestinal leakage.

## 5. Conclusions

Soft feces are rich in beneficial microorganisms (*Muribaculaceae*, *Ruminococcaceae_NK4A214_group*, and *Christensenellaceae_R-7_group*) and their metabolites (SCFAs and cytokines). Feeding on soft feces plays a very important role in improving the immune microenvironment of the cecum.

## Figures and Tables

**Table 1:** The composition and nutritional composition of diets (dry basis).

Feeds	Contents (%)	Nutrient level	Contents (%)
Soybean meal	20.0	ME (MJ/kg)^(2)^	12.20
Corn	16.0	Crude protein	14.88
Wheat bran	20.0	Crude fiber	16.53
Alfalfa meal	15.0	Ether extract	2.64
Peanut	24.5	Met	0.65
Soybean oil	0.5	Lys	0.98
Premix^(1)^	4.0	Ca	1.05
Total	100.0	P	0.35

(1) Premix is V_A_ 8000 IU, V_D3_ 900 IU, V_E_ 100 mg, V_K3_ 2 mg, V_B1_ 1 mg, V_B2_ 3 mg, V_B6_ 1 mg, V_B12_ 0.01 mg, niacin 50 mg, pantothenic acid 8.0 mg, folic acid 0.5 mg, zinc 50 mg, iron 50 mg, manganese 30 mg, magnesium 150 mg, iodine 0.5 mg, selenium 0.1 mg, salt 5 g, choline 1.5 g, methionine 3.0 g, and lysine 2.9 g per kg of diet. (2) The metabolizable energy is the calculated value, and the rest is the measured value.

## Data Availability

The raw sequence files were deposited to the National Center for Biotechnology Information Sequence Read Archive with accession number PRJNA774036.

## Supplementary Materials

Supplementary 1 Table S1: the ASV/OTU abundance table.

Supplementary 2 Table S2: the hierarchical sample metabolic pathway abundance table.

Supplementary 3 Table S3: primer information used in this study.
