# Supplementary material for: Microbiome-Metabolome Analysis of the Immune Microenvironment of the Cecal Contents, Soft Feces, and Hard Feces of Hyplus Rabbits
Source: Oxid Med Cell Longev. 2022 Oct 30;2022:5725442. doi: 10.1155/2022/5725442 (PMC9713467; doi:10.1155/2022/5725442)
Supplement: Supplementary 3 — Table S3: primer information used in this study. [file 5725442.f3.docx]

**Appendix Table 3 Primer information used in this study**

| Gene | Forward primer | Reverse primer | Length |
| --- | --- | --- | --- |
| *ZO-1* | TCCATAGAGACCGGCGTCA | GGTTTTAGGATCACAGTGTGGC | 222 bp |
| *CLDN1* | AGATGCGGATGGCTGTCAT | AAGTAGGGCACCTCCCAGAA | 202 bp |
| *OCLN* | TCCGACTTCGTGGAGAGAGT | TACTGCTGCTGCTCAAACGA | 181 bp |
| *GAPDH* | CGATGCCCCCATGTT TGTGA | TCATGAGCCCCTCCACAATG | 149 bp |
